# Efficacy and Safety of Treatment With Ferric Carboxymaltose in Patients With Cirrhosis and Gastrointestinal Bleeding

**DOI:** 10.3389/fmed.2020.00128

**Published:** 2020-04-17

**Authors:** Raquel Ballester-Clau, Gisela Torres Vicente, Mercedes Cucala Ramos, Carles Aracil Blanch, Josep Maria Miñana Calafat, Eva Pijoan Comas, Josep Maria Reñé Espinet, Montse Planella de Rubinat

**Affiliations:** ^1^Department of Gastroenterology, University Hospital Arnau de Vilanova, Lleida, Spain; ^2^Biomedical Research Institute of Lleida, Lleida, Spain; ^3^Medical Department, Vifor Pharma España SL, Barcelona, Spain

**Keywords:** liver cirrhosis, gastrointestinal bleeding (GIB), intravenous iron (i.v.), ferric carboxymaltose (FCM), blood transfusion

## Abstract

**Background:** Patients with liver cirrhosis and gastrointestinal bleeding (GIB) often develop anemia. Ferric carboxymaltose (FCM) is an intravenous (i.v.) iron formulation approved for use in patients with iron deficiency with inadequate response to oral iron therapy or when oral iron cannot be used. Here we analyzed the efficacy and safety of FCM treatment in cirrhotic patients with anemia and GIB.

**Methods:** Retrospective observational study of patients with cirrhosis and acute or chronic GIB treated with 1,000 mg FCM at the University Hospital Arnau de Vilanova (Lleida, Spain) that follows a restrictive-transfusion strategy. All data were obtained from the patients' medical records. We used the Wilcoxon test to evaluate statistical significance.

**Results:** Patients with cirrhosis and GIB (*n* = 34) were treated with 1,000 mg FCM. Portal hypertension were present in 88.2% of the patients. For hospitalized patients (*n* = 21), median serum hemoglobin (s-Hb) levels increased by 3.0 g/dL (*p* < 0.02) and 3.9 g/dL (*p* < 0.07) for patients treated with FCM who had or had not received also a transfusion, respectively, compared to levels recorded upon admission. For outpatients (*n* = 13) the mean s-Hb levels was 9.8 ± 1.6 g/dL before FCM treatment and 11.3 ± 2.1 g/dL after treatment, demonstrating a mean increase of 1.5 g/dL (*p* < 0.001). No serious adverse reactions to FCM were observed.

**Conclusion:** FCM administration achieved optimal s-Hb levels in most cirrhotic patients with acute or chronic GIB, suggesting that early FCM infusion improves and maintains optimal s-Hb levels in these patients and may be an appropriate first-line therapy to treat their anemia.

## Introduction

Liver cirrhosis is a heterogeneous condition associated with a high morbidity and economic burden (1). One of the main consequences of cirrhosis is portal hypertension, which is responsible for most cirrhotic complications and can develop long before the appearance of clinical signs (2). Portal hypertension can lead to gastrointestinal bleeding (GIB) in any part of the gastrointestinal tract. In the case of upper gastrointestinal bleeding (UGIB), the main causes are gastroesophageal variceal rupture, portal hypertensive gastro-duodenopathy, gastric antral vascular ectasia (GAVE), or peptic ulcers (3). The development of GIB, which can be acute or chronic, can cause iron-deficiency anemia (IDA), a complication present in 75% of patients with advanced liver disease (3–5). Acute UGIB is the second most common cause of mortality in patients with cirrhosis. Moreover, patients with cirrhosis develop IDA from other causes, which include hemolysis, chronic inflammatory status, or the action of drugs against hepatitis C (6, 7). IDA contributes to the poor physical and mental quality of life in these patients (8).

In clinical practice, the effectiveness of oral iron intake can be restricted by both the safety and efficacy profile of this approach. Although convenient, many patients will experience significant gastrointestinal side effects such as abdominal pain and constipation, reducing their adherence to treatment. In addition, continued treatment with oral iron can lead to black stools, which mask new GIB in these patients. Also limited absorption and poor endothelial reticulum distribution, due to their underlying condition, can inhibit optimal iron absorption. Ferric carboxymaltose (FCM) is a non-dextran i.v. iron formulation, which has been approved for use in patients with iron deficiency who have had inadequate response to oral iron therapy, or when oral iron intake cannot be used (9). Treatment with FCM is especially useful either when gastrointestinal absorption of oral iron is compromised or a fast hematopoietic response is needed. An additional beneficial effect of FCM over oral iron therapy in cirrhotic patients is that does not cause constipation, hence a lower probability of hepatic encephalopathy. FCM has shown a good efficacy and safety in patients before and after surgery, patients presenting gastrointestinal bleeding, inflammatory bowel disease, cancer or patients with chronic kidney disease or heart failure (10, 11). FCM is safer and superior to oral iron in increasing s-Hb levels in IDA with inadequate oral iron response, regardless of etiology of the anemia (12).

There are a few studies of the efficacy of i.v. FCM in acute or chronic GI bleeding anemia of different etiologies (13–17), but there are no data on its use in the treatment of anemia after acute or chronic GIB in patients with cirrhosis. Therefore, the objective of our study was to investigate the efficacy and safety of FCM treatment in cirrhotic patients with acute or chronic GIB.

## Methods

**Figure 1:**
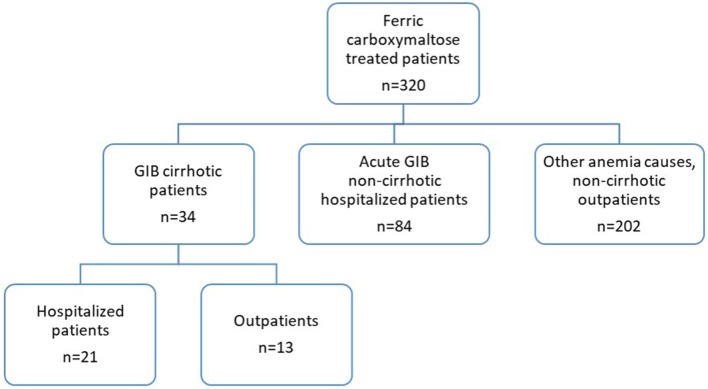
Study flowchart.

Here we present the results of a retrospective observational study of patients with liver cirrhosis and acute or chronic GIB who were treated with FCM (a single 1,000 mg dose i.v. infusion per session) at the Department of Gastroenterology of the University Hospital Arnau de Vilanova of Lleida, Spain, as routine clinical practice during the period from October 2012 to December 2015. Patients for inclusion in the current analysis were identified by searching the pharmacy records for all patients who received FCM during the study period, and then each clinical history was checked manually (Figure 1), the acute GIB non-cirrhotic patients shown in the flowchart (*n* = 84) were published previously by our group (16). The hospital follows a restrictive blood transfusion policy where blood transfusion was administered on admission or during hospitalization if the s-Hb level was lower than 7 g/dL, or at higher levels of s-Hb if comorbidities or hemodynamic instability were present (at the physician's discretion). Acute GIB was defined as visible GI bleeding (hematemesis, melena, or rectal bleeding). Chronic GIB was considered when s-Hb levels were <13 g/dL in males and <12 g/dL in females without visible GI bleeding and when other causes of anemia had been excluded (18). The following information was obtained from the patients' medical records: demographic and clinical data, portal hypertension, cause of cirrhosis, cause of hemorrhage, Child-Pugh score, Charlson comorbidity index, Meld score, medical treatment (number of blood transfusion units, total dose of FCM), s-Hb levels (at hospital admission, at discharge, and at follow-up), and length of hospital stay. Due to the retrospective nature of this study, iron metabolism parameters were only available in a few cases, and decision on iron administration was only influenced by s-Hb levels (5). The mean difference between the lowest s-Hb level at hospital admission and the s-Hb levels at hospital discharge or at the first follow-up visit were calculated.

In outpatients, the mean s-Hb level before and after FCM treatment was assessed. Values were reported as n (%) for categorical variables and as mean ± SD values for continuous variables. Values were compared between subpopulations using the χ^2^-test, Student's *t*-test, paired *t*-test, or Wilcoxon test, as appropriate.

The study was carried out in accordance with the Declaration of Helsinki and Good Clinical Practice Guidelines. The study protocol was approved by the Hospital Ethics Committee and Health Authorities (protocol number: RBC-HIE-2018-01).

## Results

In a 3-year period, from October 2012 to December 2015, 34 patients with cirrhosis and GIB were treated with FCM (Figure 1). The basal clinical characteristics of the patients are shown in Table 1. Most patients were men (64.7%), the mean ± SD age was 64.7 ± 13.1 years, and the main cause of cirrhosis was alcohol abuse (61.8%). Patients with acute GIB were on average younger than patients with chronic GIB (*p* < 0.001). Clinical, ultra-sonographic and endoscopic data of portal hypertension were observed in 88.2% of the patients. In total, 63 courses of treatment with FCM were administered, 38% upon hospital admission, and 62% in follow-up visits. Most patients (79%) received 1–2 treatment courses of 1,000 mg, the rest (21%) received 3–4 treatment courses, and one patient up to 8 courses.

**Table 1:** Patient baseline clinical characteristics (*n* = 34).

	**Total (*n* = 34)**	**Acute GIB (*n* = 15)**	**Chronic GIB (*n* = 19)**
Sex (men); *n* (%)	22 (64.7)	11 (73.3)	11 (57.9)
Age (years) mean ± SD^1^	64.7 ± 13.1	55.2 ± 8.4	72.3 ± 11.0
Cause of cirrhosis; *n* (%)			
Alcohol	21 (61.8)	11 (73.3)	10 (52.6)
HBV or HCV	8 (23.5)	2 (13.4)	6 (31.6)
Other	5 (14.7)	2 (13.3)	3 (15.8)
Child-Pugh score; mean ± SD	6.53 ± 1.4	6.67 ± 1.3	6.42 ± 1.5
A [*n* (%)]	17 (50.0)	8 (53.4)	9 (47.4)
B [*n* (%)]	15 (44.1)	5 (33.3)	10 (52.6)
C [*n* (%)]	2 (5.6)	2 (13.3)	0 (0.0)
Charlson comorbidity index; mean ± SD	6.24 ± 2.7	5.47 ± 2.3	6.84 ± 2.9
score 3–4; *n* (%)	11 (32.4)	5 (33.3)	6 (31.6)
score ≥5; *n* (%)	23 (67.6)	10 (66.7)	13 (68.4)
Portal hypertension^2^; *n* (%)	30 (88.2)	14 (93.3)	16 (84.2)
MELD score; mean ± SD	9.67 ± 2.9	10.40 ± 3.9	9.06 ± 1.5

*Values expressed as n (%) unless otherwise stated*.

1 *Statistical significance between acute GIB and chronic GIB p < 0.001*.

2 *Patients fulfilled clinic, biochemistry, radiographic and/or endoscopic criteria for portal hypertension*.

*HBV, hepatitis B virus and HCV, hepatitis C virus; SD, standard deviation; GIB, gastrointestinal bleeding; Meld score: Model for End-stage Liver Disease*.

Of 34 patients treated with FCM, 21 were admitted to the hospital. Two of them were admitted twice, so a total of 23 hospital admissions were registered for the 21 patients. In each admission, all patients received one FCM course, except a single patient that received two courses of FCM at the same admission. Therefore, a total of 24 courses of FCM were administered over 23 admissions. Of the 21 admitted patients, 14 (16 admissions) were due to acute GIB (s-Hb = 7.26 ± 1.14 g/dL, as the lowest s-Hb level) and 7 due to chronic GIB (s-Hb = 6.68 ± 1.99 g/dL, as the lowest s-Hb level).

**Figure 2:**
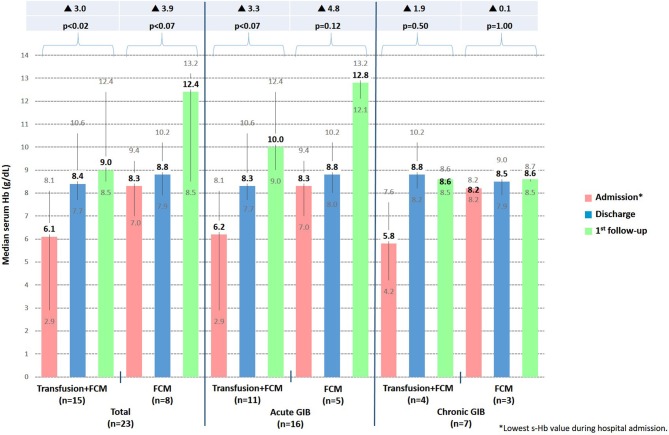
Progression of median s-Hb levels at admission, discharge and at first visit follow-up in all 23 hospital admissions (*n* = 21 patients). FCM, ferric carboxymaltose; s-Hb, serum hemoglobin; GIB, gastrointestinal bleeding.

The characteristics of the admitted patients and the cause of GIB and treatment are shown in Table 2. The most frequent cause of admission was bleeding due to esophageal varices, which was observed in 77.3% of patients. In all cases, a 1,000 mg single dose i.v. infusion of FCM was administered upon admission, and 15 (65.2%) also required a red blood cell transfusion. As shown in Table 2, the number of blood transfusions administered during admissions was similar in acute and chronic patients. When a transfusion was administered in addition to FCM the mean hospital stay was (mean ± SD) 12.2 ± 8.3 days, and when only FCM was administered the mean hospital stay was of 8.8 ± 3.1 days. Five patients received extra infusion of FCM after discharge (4 patients 1 course, 1 patient 3 courses). Serum Hb levels were recorded at hospital admission (lowest s-Hb), at hospital discharge and at the first follow-up visit 2.5–3 months later. As shown in Figure 2, s-Hb levels increased for all patients admitted. At the follow-up visit the median s-Hb levels had increased by 3.0 g/dL (*p* < 0.02) and 3.9 g/dL (*p* < 0.07) for patients who had or had not received a transfusion, respectively, compared to levels recorded upon admission. No serious adverse reactions to FCM were observed.

**Table 2:** Patients admitted to the hospital: clinical characteristics (*n* = 21 patients) and blood transfusions plus FCM treatment (*n* = 23 admissions).

	**Total (*n* = 21)**	**Acute GIB (*n* = 14)**	**Chronic GIB (*n* = 7)**	***p***
Sex (men) (%)	14 (66.7)	10 (71.4)	4 (57.1)	
Age (years); mean ± SD	61.81 (12.9)	55.41 (8.6)	74.63 (10.3)	<0.001
Portal hypertension; *n* (%)	19 (90.5)	13 (92.9)	6 (85.7)	
Causes of hemorrhage^1^; *n* (%)				
Esophageal varix	17 (77.3)	14 (93.3)	3 (42.9)	<0.05
Gastric ulcer	4 (18.2)	4 (26.7)	0 (0.0)	
GAVE (watermelon stomach)	4 (18.2)	1 (6.7)	3 (42.9)	
Other	2 (9.1)	1 (6.7)	1 (14.3)	
Child Pugh Score; mean ± SD	6.7 ± 1.4	6.5 ± 1.2	7.14 ± 1.7	
A [*n* (%)]	10 (47.6)	8 (57.1)	2 (28.6)	
B [*n* (%)]	11 (52.4)	6 (42.9)	5 (71.4)	
MELD score; mean ± SD	9.95 ± 3.3	10.21 ± 4.0	9.43 ± 1.4	
Charlson comorbidity index; mean ± SD	6.24 ± 2.7	5.36 ± 2.3	8 ±2.8	<0.04
score 3-4; *n* (%)	6 (28.6)	5 (35.7)	1 (14.3)	
score ≥5; *n* (%)	15 (71.4)	9 (64.3)	6 (85.7)	
Days in hospital; mean ± SD	11.24 ± 7.4	13.36 ± 8.0	7.00 ± 3.4	0.062
**Blood transfusions plus FCM treatment during admissions (*****n*** **=** **23)**				
	**Total (*****n*** **=** **23)**	**Acute GIB (*****n*** **=** **16)**	**Chronic GIB (*****n*** **=** **7)**	
Blood transfusion while in hospital				
Yes; *n* (%)	15 (65.2)	11 (68.8)	4 (57.1)	
•No. of blood bags; mean ± SD	3.4 ± 1.7	3.5 ± 1.9	3.3 ± 1.0	
•s-Hb (g/dL) prior to transfusion; mean ± SD	6.1 ± 1.5	6.1 ± 1.5	6.1 ± 1.8	
FCM dose per course of treatment (1,000 mg)	23 (100%)	16 (100%)	7 (100%)	

*n = 23 admissions of 21 patients*.

*Values expressed as n (%) unless otherwise stated*.

1 *more than one cause of bleeding could occur in one patient*.

*FCM, ferric carboxymaltose; GAVE, gastric antral vascular ectasia; s-Hb, serum hemoglobin; SD, standard deviation; GIB, gastrointestinal bleeding; Meld score: Model for End-stage Liver Disease*.

Thirteen out of the 34 (38.2%) cirrhotic patients with GIB did not require hospital admission and were treated with FCM. We did not hospitalized these patients if they were stable and had an s-Hb level >9 g/dL. The causes of bleeding and treatment of these patients are shown in Table 3. Only 3 patients were transfused before the administration of FCM, when the observational period started (Table 3). All patients received at least two FCM courses during the follow-up period. In summary, there were 6 patients that received 2 courses; 2 patients that received 3 courses; 4 patients that received 4 courses and one patient that received 8 courses of 1,000 mg FCM during the follow-up period. For these outpatients, the mean (± SD) s-Hb level was 9.8 ± 1.6 g/dL before FCM treatment and 11.3 ± 2.1 g/dL after treatment, demonstrating a mean increase of 1.5 g/dL during the follow-up period (*p* < 0.001). During the follow-up period the treatment courses with FCM maintained s-Hb levels around 11 g/dL avoiding transfusions in all patients during that period. There were no serious side effects resulting from FCM administration.

**Table 3:** Clinical characteristics of outpatients (*n* = 13).

	**Total (*n* = 13)**	**Acute GIB (*n* = 1)**	**Chronic GIB (*n* = 12)**
Sex (men) (%)	8 (61.5)	1 (100)	7 (58.3)
Age (years); mean ± SD	69.5 ± 12.4	51.5	71.0 ± 11.6
Portal hypertension; *n* (%)	11 (84.6)	1 (100)	10 (83.3)
Causes of hemorrhage; *n* (%)	13 (100)	1 (7.7)	12 (92.3)
Gastropathy	1 (7.7)		1 (8.3)
Occult/uncertain origin	5 (38.5)		5 (41.7)
Multifactorial	4 (30.8)	1 (100)	3 (25.0)
Colon cancer	2 (15.4)		2 (16.7)
Angiodysplasia	1 (7.7)		1 (8.3)
Child Pugh Score; mean ± SD	6.2 ± 1.5	9.0	6.0 ± 1.3
A [*n* (%)]	7 (53.8)		7 (58.3)
B [*n* (%)]	6 (46.2)	1 (100)	5 (41.7)
MELD score; mean ± SD	9.2 ± 1.9	13.0	8.82 (1.5)
Charlson comorbidity index; mean ± SD	6.2 ± 2.7	7.0	6.2 ± 2.8
score 3-4; *n* (%)	5 (38.5)		5 (41.7)
score ≥5; *n* (%)	8 (61.5)	1 (100)	7 (58.3)
Blood transfusion^1^			
Yes; n (%)	3 (23.1)	1 (100)	2 (16.7)
No. of blood bags (mean ± SD)	2 ± 0	2 ± 0	2 ± 0
• s-Hb (g/dL) prior to transfusion (mean ± SD)	6.5 ± 1.3	7.0	6.3 ± 1.2
FCM dose per course of treatment (1,000 mg)^2^	13 (100%)	1 (100%)	12 (100%)

*n = 13, several patients received more than one FCM course along the follow-up*.

1 *Blood transfusions were administered only once to 3 patients prior to FCM during the whole observational period*.

2 *Patients received 1,000 mg in each administration course. All patients received at least two FCM courses during the observational period. In summary, there were 6 patients that received 2 courses; 2 patients that received 3 courses; 4 patients that received 4 courses and one patient that received 8 courses*.

*FCM, ferric carboxymaltose; s-Hb, serum hemoglobin; SD, standard deviation; GIB, gastrointestinal bleeding*.

## Discussion

We report here our clinical observation of the efficacy of FCM treatment in patients with cirrhosis and anemia due to acute or chronic GIB. In patients admitted to hospital, we observed a significant s-Hb level increase at discharge and at follow up, regardless of whether patients received transfusion plus FCM or only FCM. Which means that when a restrictive-transfusion strategy is used, only the use of FCM infusion achieves an optimal s-Hb level. Obviously, patients who received transfusion plus FCM had significantly lower initial s-Hb than patients who only received FCM, therefore the increase of s-Hb was higher in the first ones during hospitalization. But looking at the s-Hb increase from admission to first follow-up, patients receiving FCM alone had a higher s-Hb increase. Outpatients, also showed a satisfactory s-Hb response and those patients who needed subsequent doses of FCM during the follow-up period maintained acceptable s-Hb levels without requiring transfusion.

Recently, a randomized clinical trial (RCT) showed that red blood cell transfusion improved survival in patients with cirrhosis and GIB when s-Hb levels dropped below 7 g/dL (restrictive strategy), probably because of a better control of further bleeding, as restitution of blood volume can increase portal hypertension (19). In these cases, administration of i.v. iron can reduce the amount of red blood cell transfusions needed, helping to overcome IDA (20). In addition, transfusion should be administered cautiously, as other comorbidities, such as ischemic heart disease, could influence the outcome (21). Blood transfusions raise s-Hb levels only transiently without correcting iron depletion at the same time, making future anemia more likely (22). In this regard, i.v. iron allows the repletion of iron stores and erythropoiesis (15, 23). For example, FCM can prevent recurrence of anemia in patients with inflammatory bowel disease, compared with placebo (24).

When acute and chronic bleeding were analyzed separately, the highest rise in s-Hb levels were observed among patients with acute bleeding, either hospitalized or not. As shown in Table 2, patients with chronic bleeding had a higher absolute score in the Charlson scale. It means that they presented more comorbidities, which can lead to other causes of anemia different from hemorrhage. It should be noted that 67% of our patients had a Charlson comorbidity index ≥5 and 50% had a Child- Pugh between B and C, especially those with chronic GIB, who also were 72 years of age on average (Table 1). Despite these parameters, this population presented an optimal s-Hb increase and a good safety profile.

In our study we did not detect any serious adverse reaction. A recent review by the European Medicines Agency, based in part on information from 6,608 patients who had received FCM, concluded that the benefits of i.v. iron outweigh the risks of treating IDA when the oral route is insufficient or poorly tolerated (25). Furthermore, i.v. iron treatment usually contributes to faster iron recovery than oral treatments (10, 15, 22).

To our knowledge, this is the first report of FCM administration in patients with liver cirrhosis presenting GIB. The evidence for the use of i.v. iron in patients with GIB and IDA is limited, however recent studies suggest its clinical efficacy in related conditions (10, 12, 14–16, 20, 24). Intravenous iron has also been successfully used for treating of anemia in patients with colon cancer and other surgical procedures (13, 26). Recently, two studies have shown the efficacy and safety of FCM in patients with acute upper or lower GIB. A single 1000 mg i.v. dose of FCM in patients with acute GIB, as part of a restrictive-transfusion policy, is associated with a good erythropoietic response and recovery from anemia after the immediate period of hospitalization (16). Although it is not the total recommended dose to replenish iron deposits, a single infusion of FCM 1,000 mg is enough to induce an adequate erythropoietic response in at-risk subgroups including senile patients, those with high comorbidity, and patients with moderate or severe anemia. Almost normal s-Hb levels were achieved in these three groups, in whom resolving anemia may be particularly important. FCM therapy was safe and well tolerated in these patients with no severe adverse events reported. Providing effective iron repletion during hospitalization through FCM therapy may help to avoid or reduce blood transfusion requirements even in high-risk patients and the anemic syndrome so frequently observed after acute GIB (16). Ferrer-Barceló et al., in a RCT that compared i.v. FCM vs. oral ferrous sulfate in patients with anemia after acute non-variceal GIB, demonstrated that FCM offers a faster and more efficient normalization of s-Hb levels and iron repletion stores compared to oral iron therapy (15). Additionally, the administration of FCM is associated with few adverse events and improved patient quality of life, further contributing to an overall favorable risk-benefit profile (15). Overall, the results of the previous studies and the one presented here, provide clinical support for considering patients with anemia secondary to acute or chronic GIB regardless of its origin (cirrhotic or not), can benefit from FCM treatment and to include FCM into treatment schemes and guidelines.

The main limitation of our study is that it is a single-center retrospective descriptive analysis based on the center's regular clinical practice; secondly, markers of iron status were available infrequently in the patient's records and thirdly, the relatively small number of patients included since it is a medium-sized hospital. Due to these limitations, the results must be judged with caution.

In conclusion, the present study suggests that early FCM treatment may improve, maintain and preserve s-Hb levels in cirrhotic patients with IDA secondary to acute or chronic GIB. Further prospective RCT are warranted to ascertain whether FCM may be an appropriate first-line treatment of IDA in these patients, allowing for the extensive use of a restrictive-transfusion strategy.

## Data Availability Statement

All datasets generated for this study are included in the article/supplementary material.

## Ethics Statement

The studies involving human participants were reviewed and approved by University Hospital Arnau de Vilanova of Lleida. Written informed consent for participation was not required for this study in accordance with the national legislation and the institutional requirements.

## Author Contributions

RB-C, GT, CA, JM, EP, JR, and MP managed the patients and collected the data. MC provided medical support. All authors critically analyzed the data, wrote and reviewed the manuscript, and approved the final version for publication.

## Conflict of Interest

MC is an employee of Vifor Pharma España S.L. The remaining authors declare that the research was conducted in the absence of any commercial or financial relationships that could be construed as a potential conflict of interest.
